# Achievement of High-Response Organic Field-Effect Transistor NO_2_ Sensor by Using the Synergistic Effect of ZnO/PMMA Hybrid Dielectric and CuPc/Pentacene Heterojunction

**DOI:** 10.3390/s16101763

**Published:** 2016-10-21

**Authors:** Shijiao Han, Jiang Cheng, Huidong Fan, Junsheng Yu, Lu Li

**Affiliations:** 1 State Key Laboratory of Electronic Thin Films and Integrated Devices, School of Optoelectronic Information, University of Electronic Science and Technology of China (UESTC), Chengdu 610054, China; 201211050108@std.uestc.edu.cn (S.H.); 201511050103@std.uestc.edu.cn (H.F.); 2 Co-Innovation Center for Micro/Nano Optoelectronic Materials and Devices, Research Institute for New Materials and Technology, Chongqing University of Arts and Sciences, Chongqing 402160, China; caiwu0301@163.com

**Keywords:** NO_2_ sensors, organic field-effect transistor (OFET), synergistic effect, ZnO/PMMA hybrid dielectric, heterojunction structure

## Abstract

High-response organic field-effect transistor (OFET)-based NO_2_ sensors were fabricated using the synergistic effect the synergistic effect of zinc oxide/poly(methyl methacrylate) (ZnO/PMMA) hybrid dielectric and CuPc/Pentacene heterojunction. Compared with the OFET sensors without synergistic effect, the fabricated OFET sensors showed a remarkable shift of saturation current, field-effect mobility and threshold voltage when exposed to various concentrations of NO_2_ analyte. Moreover, after being stored in atmosphere for 30 days, the variation of saturation current increased more than 10 folds at 0.5 ppm NO_2_. By analyzing the electrical characteristics, and the morphologies of organic semiconductor films of the OFET-based sensors, the performance enhancement was ascribed to the synergistic effect of the dielectric and organic semiconductor. The ZnO nanoparticles on PMMA dielectric surface decreased the grain size of pentacene formed on hybrid dielectric, facilitating the diffusion of CuPc molecules into the grain boundary of pentacene and the approach towards the conducting channel of OFET. Hence, NO_2_ molecules could interact with CuPc and ZnO nanoparticles at the interface of dielectric and organic semiconductor. Our results provided a promising strategy for the design of high performance OFET-based NO_2_ sensors in future electronic nose and environment monitoring.

## 1. Introduction

Since air pollution has become an urgent global problem with the development of industry and technology, detecting gases, especially toxic gases, as the basis for controlling air pollution, has become increasingly significant [1]. One of the most common and detrimental air pollutant oxidizing gases is nitrogen oxides, including nitrogen dioxide (NO_2_), which is produced and released into atmosphere from combustion and automotive emission. In addition to contributing to the formation of fine particle pollution, NO_2_ is linked with a number of adverse effects on the respiratory system such as chronic bronchitis, emphysema, and respiratory irritation at low concentrations [2,3,4]. The potential detrimental impact of NO_2_ emission on public health and the environment has led to extensive scientific and technological progress in the field of NO_2_ sensors. Therefore, many NO_2_ sensors are commercially available, such as electrochemical, resistive, and optical sensors [5,6]. Optical methods, which rely on the unique optical fingerprints of NO_2_ gas molecules, have the highest sensitivity despite their sizes and costs [7]. Electrochemical sensing mainly depends on electrochemical reduction between NO_2_ and catalysts, which is low-cost but has a short lifetime [8]. NO_2_ sensing based on resistive relies on the charge transfer between metal oxides and NO_2_ absorbed on the surface, which has poor selectivity and needs high temperature to achieve recovery or reaction [9]. However, with the development of two-dimensional (2D) materials, Ou et al. have realized selective and reversible NO_2_ gas sensing by using the charge transfer between physisorbed NO_2_ gas molecules and 2D tin disulfide (SnS_2_) and molybdenum disulfide (MoS_2_) at low operating temperatures [10,11].

As a new developing sensing platform, organic field-effect transistor (OFET)-based sensors have attracted intriguing attention owing to their advantages of plenty organic material resources, mechanical flexibility, and microarray compatibility [12,13]. As a key functional layer of an OFET-based sensor, organic semiconductor (OSC) materials have become promising candidates for gas sensors due to their high sensitivity, low production costs, and room temperature detection [14,15]. Mostly, the efforts involved in developing a high performance OFET-based gas sensor are mainly focused on the OSC layers [16]. However, the interface property of dielectrics also plays a crucial role in the gas sensing characteristics, as the efficient current channel lies in the first few molecular layers of the OSC upon the dielectric layer [17]. Using scanning Kelvin probe microscopy, Andringa et al. have determined that the trapped electrons of OFET-based sensors are located at the dielectric interface [18]. Therefore, modifying dielectrics with certain functional materials could significantly improve the sensing properties of OFET-based chemical sensors.

Furthermore, great efforts in device engineering of OFETs have been made to construct a series of sensing devices with high selectivity, one of the most efficient methods is to implant functional receptors [14]. Specifically, metal phthalocyanines (MPc) molecules have low ionization energy, which imparts low activation energy for the formation of charge transfer complexes with oxidizing gases [13]. NO_2_ is a strong-binding gaseous oxidant, so MPc films have been widely used in NO_2_ detection, such as copper(II) phthalocyanine (CuPc), titanyl phthalocyanine (TiOPc) and hexadecafluorinated copper phthalocyanine (F16CuPc) [19]. Yan et al. reported a TiOPc/F16CuPc heterojunction gas sensor with an enhanced relative response to NO_2_ as low as 5 ppm and a detection limit down to 250 ppb at room temperature [20]. By adding a highly sensitive vanadyl phthalocyanine (VOPc) layer on top of the single heterojunction device to form a double heterojunction, the relative-response intensity could be improved significantly [21]. This result indicated that using a MPc ultrathin heterojunction can present a high response NO_2_ gas sensor, which makes it very promising in the low-cost room-temperature-sensor area. As our previous research shows, zinc oxide (ZnO) nanoparticles at the OSC/dielectric interface would decrease the grain size of OSC deposited on the hybrid dielectric, and, meanwhile, the boundary between crystals was increased [22]. More notably, ZnO and related nanostuctures, suh as NO_2_ sensible materials, have extensively been used in resistance and field-effect transistor-based NO_2_ sensors [23,24,25]. However, the work on the use of synergistic effect of ZnO nanoparticles and CuPc/pentacene heterojunction to enhance the relative response of the OFET-based gas sensors was rarely reported. Therefore, our research can develop a new strategy to realize the high relative response commercial OTFT-based gas sensors.

In this work, we used the synergistic effect of zinc oxide/poly(methyl methacrylate) (ZnO/PMMA) hybrid dielectric and CuPc/pentacene heterojunction to realize high sensitivity OFET-based NO_2_ gas sensors. The ZnO/PMMA hybrid dielectric was fabricated by simply blending the ZnO nanoparticles and PMMA solutions, and the heterojunction was formed by adding an ultrathin CuPc layer on the top of pentacene. The synergistic effect of hybrid dielectric and organic semiconductor layers was characterized by the control experiments. Moreover, the selectivity and stability of this OFET-based gas sensor were characterized in detail. The results of this research may improve our understanding of design strategy for OFET-based gas sensors.

## 2. Materials and Methods

### 2.1. ZnO/PMMA Hybrid Preparation

ZnO nanoparticles were prepared according to the method reported in the previous literature [26], and the average size of as-synthesized quasispherical ZnO NPs was 4.9 nm. ZnO nanoparticles were divided from methanol by centrifugation and dispersed in chloroform/methanol (50 mL, v/v = 90:10) to obtain a stock solution. PMMA (average molecules weight ~ 120,000) was dissolved in anisole with a concentration of 200 mg/ml. The obtained solution was mixed with the prepared ZnO nanoparticles dispersion (v/v = 1:1). The ZnO/PMMA hybrid dielectric was fabricated using spin coating process.

### 2.2. Device Preparation

**Figure 1:**
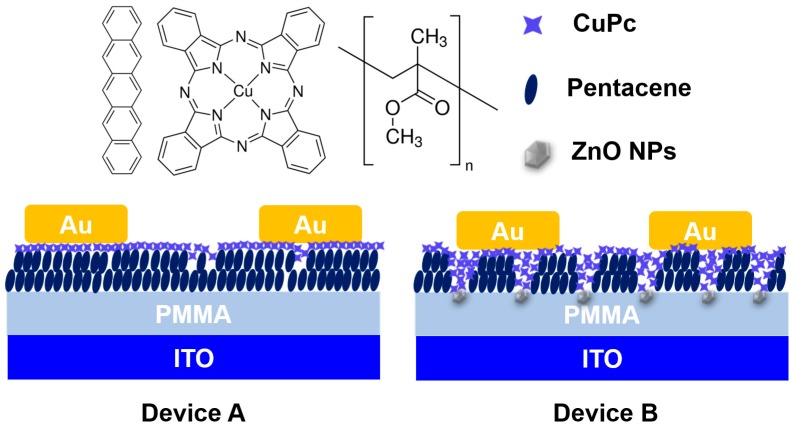
Molecular structures of the pentacene, CuPc and PMMA, along with the organic field-effect transistor (OFET)-based sensor device configurations in this study, device A with only CuPc/Pentacene heterojunction; device B with both ZnO/PMMA hybrid dielectric and CuPc/Pentacene heterojunction.

Figure 1 shows the molecular structures of pentacene, CuPc and PMMA and schematic structure of the top-contacted OFET-based sensors with only CuPc/pentacene heterojunction (device A) and both ZnO/PMMA hybrid dielectric and CuPc/pentacene heterojunction (device B). The OFETs were processed according to the following procedure. Indium tin oxide (ITO) coated glass was used as substrate and gate electrodes. Prior to the spin-coating of the dielectric layers, the substrates were successively ultrasonically cleaned in acetone, deionized water and isopropyl alcohol. ZnO/PMMA hybrid and PMMA, functioned as the gate dielectric, were spin-coated on ITO substrate at room temperature (25 °C) and baked in an oven at 90 °C for 2 h. Subsequently, 30 nm pentacene and 5 nm thick CuPc were thermally evaporated in a vacuum of ~2 × 10^−4^ Pa at a rate of 0.2 Å/s successively. Finally, the source and drain electrodes of 50 nm gold (Au) were thermally deposited using a shadow mask at a rate of 10 Å/s. The length and width of the channel were 100 µm and 1 cm, respectively.

### 2.3. Device Test and Data Analyses

The electrical characteristics of all the devices were measured with a Keithley 4200 sourcemeter (Tektronix, Shanghai, China) under ambient conditions at room temperature. 

The morphologies of the organic semiconductor were characterized with atomic force microscopy (AFM) (Agilent, AFM 5500) in a tapping mode. The OFET sensor was stored in an airtight test chamber (approximately 0.02 L). Dry air and 100 ppm standard NO_2_ gases (anhydrous) were purchased from Sichuan Tianyi Science and Technology Co., Chengdu, China, and a mixture with the appropriate concentration was introduced into the test chamber by a mass flow controller (S48 300/HMT, Beijing Boriba Metron Instruments Co., Beijing, China). NO_2_ gas response characteristics of the OFET sensors were measured with a variation of drain-source current, which acted as a function of time. Also, the transfer curves in various concentrations of NO_2_ were systematically characterized.

The field-effect mobility of device was extracted in the saturation regime from the highest slope of |*I_DS_*|^1/2^ vs. *V_G_* plots by using Equation (1):

(1) $$I_{DS}=(\frac{WC_{i}}{2L})\mu {\left(V_{G}-V_{T}\right)}^{2}$$

where *L* and *W* are the channel length and width, respectively. *C_i_* is the capacitance (per unit area) of the dielectric, *V_G_* is the gate voltage, and *I_DS_* is the drain-source current.

## 3. Results and Discussion

**Figure 2:**
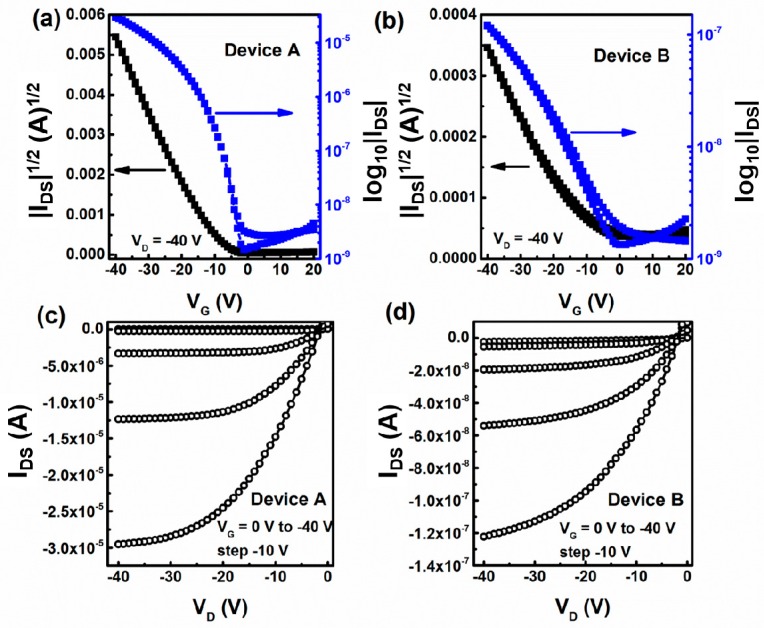
(**a**,**b**) Typical transfer curve I_DS_-V_G_, and (**c**,**d**) output curve I_DS_-V_D_ of devices A and B, respectively.

Figure 2 depicts the representative transfer plots of devices A and B. Both devices A and B have the typical behavior of a p-type transistor. Device A exhibits a field-effect mobility (μ), a current on/off ratio (I_on_/I_off_), a threshold-voltage (V_T_), and a sub-threshold slope(SS) of 0.13 cm^2^·V^−1^·s^−1^, 2.0 × 10^3^, −12 V, and 3.0 V/dec, respectively. In contrast, the values of μ, I_on_/I_off_, V_T_, and SS for device B are 0.006 cm^2^·V^−1^·s^−1^, 8.9 × 10^1^, −15 V and 15 V/dec, respectively. It is obvious that the device performance of CuPc/pentacene heterojunction OFETs based on pure PMMA is much higher than that of based on ZnO/PMMA hybrid dielectric. Compared with our previous results [22], ZnO nanoparticles can lead to more serious performance decrease on CuPc/pentacene heterojunction-based device than the pentacene-based device. This phenomenon indicates that CuPc may also play an essential role as well as ZnO nanoparticles, i.e., they have a synergistic effect.

**Figure 3:**
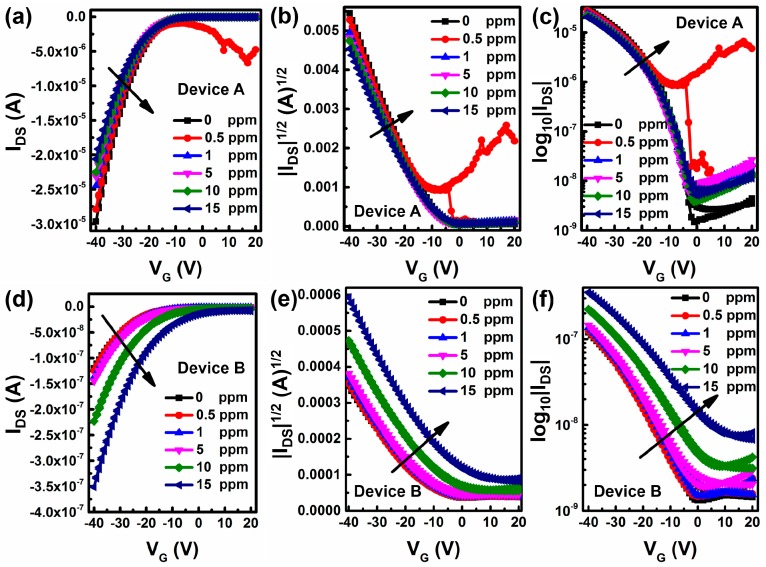
Transfer curves of devices A and B under a specific concentration of NO_2_, (**a**,**d**) without calculation, (**b**,**e**) after taking log, (**c**,**f**) after extracting.

Then, the OFETs were exposed to NO_2_ atmosphere with various concentrations that ranged from 0 to 15 ppm. All the devices were exposed to a specific concentration of NO_2_ for 5 min before measuring. The gate voltage *V_G_* was from 20 to −40 V and the drain voltage *V_D_* was −40 V. As shown in Figure 3, the curves of device A exhibit a slight shift, but that of device B shifts more significantly.

**Figure 4:**
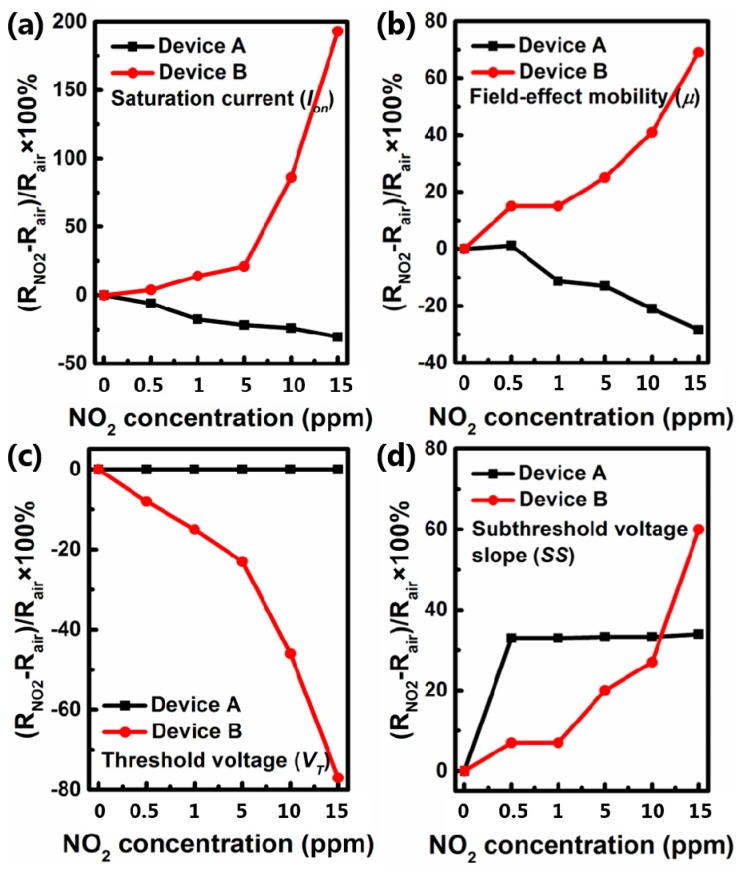
Percentage variation of I_on_ (**a**), μ (**b**), V_T_ (**c**) and SS (**d**) of all the devices at different NO_2_ concentrations, respectively.

To intuitively illuminate sensing property, several parameters were calculated from the transfer curve to evaluate the performance of OFET sensors, such as I_on_, μ, V_T_, and SS. The variation of multiple parameters defined as ΔR = (R_NO2_ − R_AIR_)/R_AIR_ × 100% is presented in Figure 4. As shown in Figure 4a,b, the variations of I_on_ and μ of device A and device B show an opposite trend along with the increasing concentration of NO_2_, the I_on_ and μ of device B increase by 193% and 69% at 15 ppm NO_2_, while that of device A decreases by 30% and 28%. Since the I_on_ and μ of pentacene-based OFETs will increase significantly when exposed to NO_2_ atmosphere [17], CuPc might have a different impact on devices A and B. The V_T_ of device B presents a remarkable decrease about 80%, while that of device A shows nearly no change. Because V_T_ is usually referred to charge trapping at the dielectric/semiconductor interface, the more hole charges trap at the interface, the stronger negative gate voltage is needed to turn the transistor on, and vice versa. Thus, the dielectric/semiconductor interface of device A has less trap sites than device B after being exposed to NO_2_, and the NO_2_ interact places do not locate at this interface. 

Furthermore, *SS* is proportional to the trap density at the interface of dielectric and organic semiconductor, and the trap density (N) can be extracted by Equation (2):

(2) $$SS=\frac{kT}{q}\log_{10}(1+\frac{qN}{C}{)}^{2}$$

where *q* is the electronic charge, *k* is Boltzmann’s constant, *T* is absolute temperature, and *C* is the areal capacitance of the dielectric structure. So the *SS* is proportional to the *N*. As shown in Figure 4d, the *SS* increases constantly to 60% in device B at 15 ppm NO_2_ concentration. Nevertheless, the SS of device A is almost unchanged under the concentration of 0.5–15 ppm NO_2_. So the interaction in device A between NO_2_ and dielectric/organic semiconductor interface is different from that of device B.

**Figure 5:**
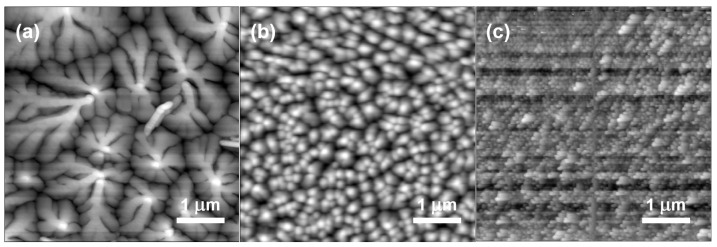
Atomic force microscopy (AFM) topography images of the pentacene films on pure PMMA dielectric (**a**); ZnO/PMMA hybrid dielectrics (**b**) and CuPc film on it (**c**).

To study the reason for the deviation in sensing performance, AFM was utilized to observe the morphologies of active films (AFM images of dielectrics are shown in Figure S1). As shown in Figure 5a,b, the grain size of pentacene in device B is much smaller than that in device A, indicating that ZnO nanoparticles embedded in PMMA dielectric act as impurities and influence the morphology of pentacene film by three aspects: decreasing the grain size, deepening the grain boundary, and disordering molecular arrangement [27]. From Figure 5c, it can be clearly observed that the ultrathin film of CuPc in device B forms a relatively homogeneous film. 

**Figure 6:**
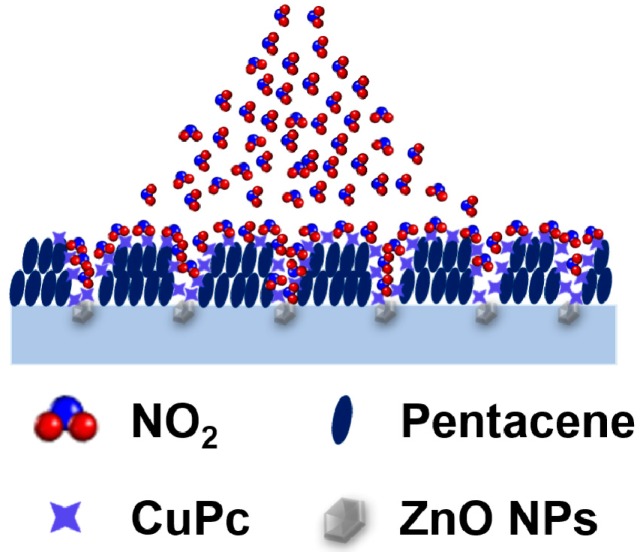
Schematic illustration of ZnO/PMMA hybrid dielectric and CuPc/pentacene heterojunction under NO_2_.

From the above discussion, it can be deduced that the enhancement of the sensing properties is attributed to the synergistic effect of ZnO/PMMA hybrid dielectric and CuPc/pentacene heterojunction: (1) ZnO nanoparticles embedded in PMMA will dramatically decrease the grain size and enlarge the depth of grain boundary, which not only increases the potential barrier but also makes the subsequent CuPc molecules partially diffuse into the interface of pentacene and hybrid dielectric. Moreover, the nitrogen atoms around the Cu atom have a relatively high electron density, which can act as hole-charge traps. Thus, the I_on_ and μ of device B are much lower than those of device A; (2) When device B is exposed to NO_2_, as shown in Figure 6, NO_2_ can diffuse directly into the interface of organic semiconductor and hybrid dielectric, then interact with ZnO nanoparticles and CuPc. As is well known, NO_2_ is a strong oxidizing gas with very high electron affinity, so it will interact with the surface of ZnO nanoparticles through surface-adsorbed oxygen ions [23]. Similarly, CuPc molecules can interact with NO_2_ and form charge complex due to the delocalized π-electrons which are readily ionized [13]. NO_2_ can weaken the impact of ZnO nanoparticles and CuPc at or near the dielectric/organic semiconductor interface on charge transport effectively, so I_on_, μ and V_T_ are significantly improved. As a result, the high relative responses can be achieved. The main difference between devices A and B is that CuPc affects the charge conducting channel directly as a functional receptor under the action of ZnO nanoparticles. Device A just forms a simple vertical heterojunction; the sensing mechanism is dependent on the energy level disordering after being exposed to NO_2_. Because the NO_2_-induced domain fracture originates at the CuPc/Au interface, it is proposed that the NO_2_-induced domain fraction also degrades the CuPc/Au electrical contacts, the increased density of domain boundaries would therefore act to trap carriers near the contacts and induce positive uncompensated charge, which is consistent with the decrease of I_on_ [13].

**Figure 7:**
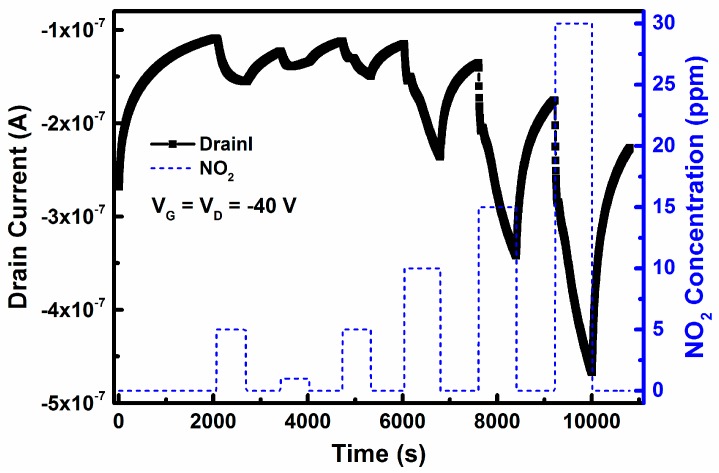
Real-time response curve of this OFET sensor based on ZnO/PMMA hybrid dielectric and CuPc/pentacene heterojunction to different NO_2_ pluses.

The real-time response curve of device B for NO_2_ detection was also studied (Figure 7). The pulse of each NO_2_ concentration was 10 min. During the recovery process, NO_2_ gas was removed, and the sensor was exposed to dry air for 10 min. It is obvious that, when exposed to different NO_2_ concentrations, device B has a fast response.

**Figure 8:**
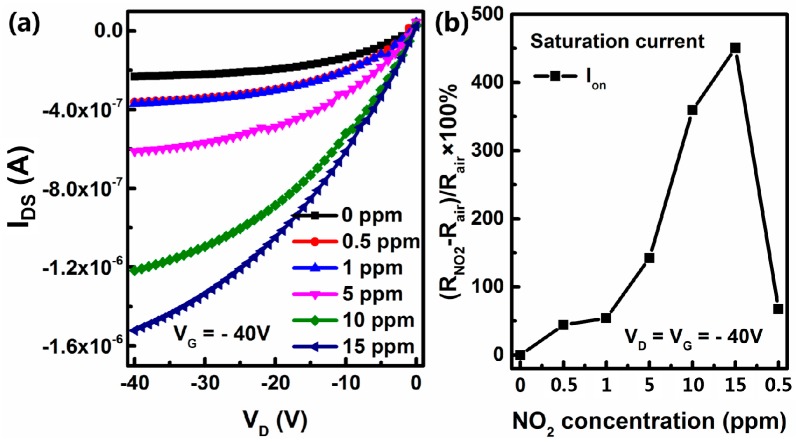
Output curve of devices B (**a**) and percentage variation of I_on_ (**b**) under a specific concentration of NO_2_ after stored under ambient for 30 days.

In addition to the response of fresh OFET sensor, the environmental stability under ambient atmosphere is of critical importance to the practical application of OFET-based sensors and the overall lifetime of the device [28,29]. Thus, the sensor that was tested was stored in ambient air with a relative humidity of ~50% for 30 days, the testing process was similar to the aforementioned fresh device characterization. The variation of its output current at V_G_ = −40 V when exposed to different NO_2_ concentrations (ranging from 0.5 to 15 ppm) is shown in Figure 8a. As shown in Figure 8b, the relative change of I_on_ at 0.5 ppm NO_2_ is more than 10 times higher than that of the fresh device (more than twice at 15 ppm). Moreover, after being exposed to 15 ppm NO_2_, the device still can detect 0.5 ppm NO_2_ effectively, and exhibit a high stability to constantly detection. As lots of H_2_O and O_2_ is absorbed on the surface of ZnO nanoparticles, a large enhancement of sensitivity may be attributed to the concerted efforts of H_2_O, O_2_ and NO_2_ [30,31,32].

**Figure 9:**
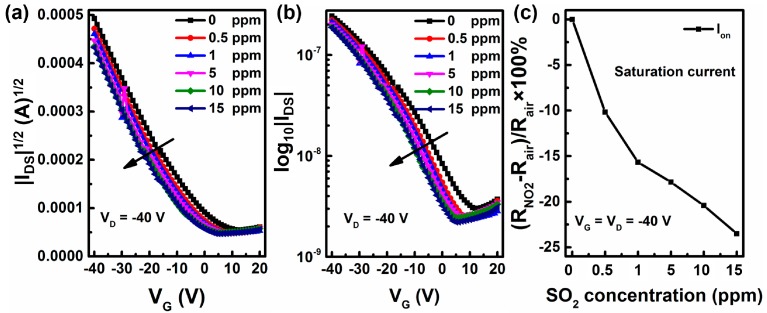
Transfer curves of devices B (**a**,**b**) and percentage variations of I_on_ (**c**) under a specific concentration of SO_2_.

Selectivity is a crucial parameter and an open issue for practical sensing applications, which usually relies on the specific interaction or energy modulation between the organic semiconductors and the analytes [33,34]. Another common kind of air pollutant oxidizing gas of sulfur oxides, sulfur dioxide (SO_2_) was also investigated by using device B. As shown in Figure 9, the V_T_ increases and the I_on_ decreases along with the increase of SO_2_ concentration. Surprisingly, this result is opposite to the conventional phenomenon of oxidizing gases [35]. This result may be due to the interaction between ZnO and SO_2_ at room temperature, yielding SO_4_^2−^, which can act as the hole trap sites at the interface of dielectric and organic semiconductor [36,37].

## 4. Conclusions

In conclusion, the ZnO/PMMA hybrid dielectric and CuPc/pentacene heterojunction were used to achieve a high response OFET-based NO_2_ gas sensor, exhibiting a dramatic variation of I_on_, *μ*, and *V_T_* after being exposed to NO_2_. The I_on_ and *V_T_* increased by 193% and 77% at 15 ppm NO_2_, and by 5% and 8% at 0.5 ppm NO_2_. Moreover, after storing at atmosphere for 30 days, the relative change of I_on_ at 0.5 ppm NO_2_ was more than 10 times higher (about 50%) than that of a fresh device. The high performance of this OFET-based sensor was attributed to the synergistic effect of ZnO/PMMA hybrid dielectric and CuPc/pentacene heterojunction. In addition, the sensor with synergistic effect could clearly distinguish the oxidizing gas of NO_2_ from SO_2_ with opposite I_on_ variation. Using the synergistic effect of dielectric and organic semiconductor is demonstrated to be an effective method for device engineering in OFET-based gas sensors.

## Supplementary Materials

The supplementary materials can be found at http://www.mdpi.com/1424-8220/16/10/1763/s1.
