# Supplementary material for: Achievement of High-Response Organic Field-Effect Transistor NO2 Sensor by Using the Synergistic Effect of ZnO/PMMA Hybrid Dielectric and CuPc/Pentacene Heterojunction
Source: Sensors (Basel). 2016 Oct 21;16(10):1763. doi: 10.3390/s16101763 (PMC5087547; doi:10.3390/s16101763)
Supplement: Supplementary file 1 [file sensors-16-01763-s001.pdf]

Supplementary Materials: Achievement of High Response Organic Field-Effect Transistor NO_2 Sensor by Using the Synergistic Effect of ZnO/PMMA Hybrid Dielectric and CuPc/Pentacene Heterojunction

Shijiao Han, Jiang Cheng, Huidong Fan, Junsheng Yu and Lu Li

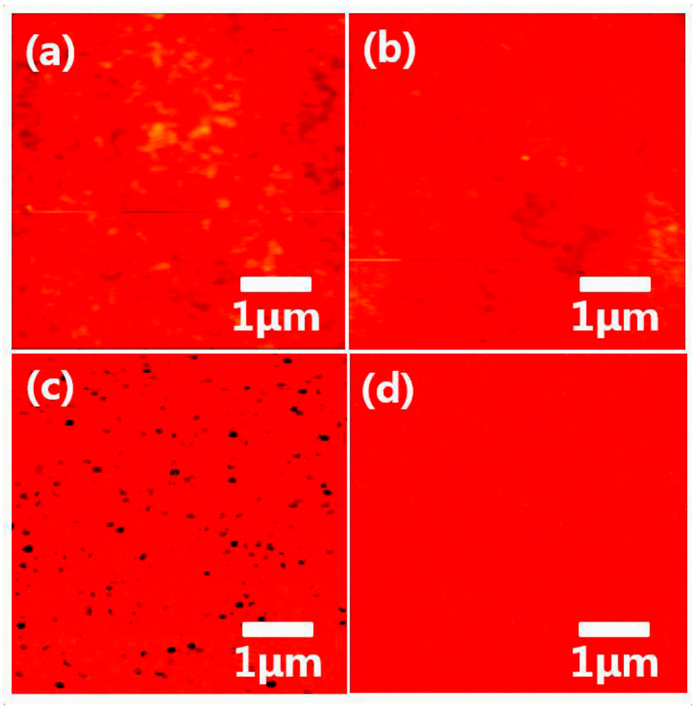

Figure S1. AFM images of ZnO/PMMA hybrid and PMMA dielectrics spin-coated on ITO glass substrate, (**a**,**b**) height images and (**c**,**d**) phase images.
